# Cell death affecting the progression of gastric cancer

**DOI:** 10.1038/s41420-022-01161-8

**Published:** 2022-08-29

**Authors:** Haoying Wang, Mengxiao Liu, Xi Zeng, Ya Zheng, Yuping Wang, Yongning Zhou

**Affiliations:** 1 The First Clinical Medical College, Lanzhou University, Lanzhou, China; 2 Department of Gastroenterology, The First Hospital of Lanzhou University, Lanzhou, China; 3 Key Laboratory for Gastrointestinal Diseases of Gansu Province, The First Hospital of Lanzhou University, Lanzhou, China

**Keywords:** Gastric cancer, Cell death

## Abstract

Gastric cancer is a gastrointestinal tumor with high morbidity and mortality rates. Several factors influence its progression, cell death being an important element. In this review, we summarized the effects of necrosis, apoptosis, necroptosis, pyroptosis, ferroptosis, and eight less common cell death modalities on gastric cancer cells and the tumor microenvironment, detailed the molecular mechanisms of various cell death and their major regulatory pathways in gastric cancer, explored the prevalence and complexity of cell death in gastric cancer progression and highlighted the potentials of cell death-related therapies in gastric cancer.

## Facts

- Cell death divided into passive necrosis and programmed cell death (PCD), and the latter is further divided into apoptosis and programmed necrosis, according to the different morphological characteristics of death.
- Necroptosis, pyroptosis, ferroptosis, and apoptosis are the most common PCDs, and they are closely related to the progression of gastric cancer.
- Eight less common PCD (MPT-driven necrosis, ADCD, LDCD, NETosis, Entosis, parthanatos, Oxeiptosis, Alkaliptosis) also had profound effects on gastric cancer and tumor microenvironment.
- PI3K/AKT/mTOR, JAK/STAT3, Keap1/Nrf2, Hypoxia/HIF-1α and other signaling pathways are involved in cell death during gastric cancer progression.
- It is a promising direction for the future treatment of gastric cancer that identifying efficient new drugs to induce gastric cancer cell death and to inhibit the death of immune cells and normal tissue cells.

## Open questions

- What are the characteristics of different types of cell death?
- What are the molecular mechanisms that different types of cell death are involved in the progression of gastric cancer?
- How to inhibit the progression of gastric cancer by interfering with cell death?

## Introduction

Gastric cancer is a common malignant tumor with the fifth-highest global incidence and fourth-highest mortality rate of all malignant tumors [1]. More than one million new cases of gastric cancer occur each year, mostly in East Asia, imposing a tremendous burden on local societies, economies, and patients’ families [1]. About 10% of gastric cancer patients showed significant familial aggregation, with germline mutations present in 1%‒3% [2]. For most patients, the pathogenesis of gastric cancer remains imprecise, although it is now generally accepted that *H. pylori* infection, smoking, alcohol consumption, a high-salt diet, and lack of exercise are independent risk factors. [3]. In recent years, genomic studies have identified mutations in HER2, FGFR2, ERBB2, ARID1A, TP53, and many other genes highly correlated with gastric cancer. Epigenetic studies revealed that post-transcriptional modifications, such as methylation and acetylation, had important effects on the progression of gastric cancer [4, 5]. Studies on non-coding RNA and exosomes have also improved the understanding of gastric cancer [5]. Interestingly, either molecular alteration ultimately influences the biological behavior of gastric cancer cells or the tumor microenvironment.

Cell death is a frequently regulated biological behavior in gastric cancer and immune cells. The earliest reported correlation between cell death and cancer dates to the 1955 study by Thomlinson and Gray on radiotherapy for lung cancer. They found that lung cancer cells irradiated with γ-radiation showed solidification and fragmentation of the nucleus and rupture of the cell membrane, indicating cell necrosis [6]. In 1972, the Australian pathologist Kerr discovered a significantly different form of cell death from necrosis: hepatocytes die after blocking portal blood flow by forming many round vesicles encased in cell membranes and encapsulating with solid chromatin [7]. Kerr named it apoptosis [7]. Many subsequent studies have found that apoptosis not only presents different morphological changes from necrosis but also occurs through distinct mechanisms. Necrosis is a passive, non-programmed form of cell death, whereas apoptosis is a strictly regulated programmed cell death (PCD) [8]. Since then, many PCDs have been identified, including necroptosis, pyroptosis, and ferroptosis. These late discoveries of PCD and apoptosis are actively regulated; however, they differ significantly from each other. Apoptotic cells do not cause an inflammatory response, whereas other PCD morphological manifestations are consistent with necrosis and are accompanied by an inflammatory response [9]. Therefore, PCD with necrotic features is uniformly classified as programmed necrosis to distinguish it from apoptosis [9].

Cell death plays a significant role in the genetic development of organisms, immune maturation, and maintenance of organismal homeostasis. Aberrant cell death is accompanied by developmental abnormalities and disease [10]. PCD dysregulation has complex effects on cancer cells and the tumor microenvironment [11, 12], suggesting the profound involvement of cell death in the progression of malignancy. In this review, we provided a detailed overview of several modes of cell death that influence gastric cancer progression; described the complex impact of necrosis, apoptosis, and programmed necrosis on tumor metabolism and therapy; and stated some of the opportunities and challenges in the field.

## Cell Death in cancer

**Fig. 1:**
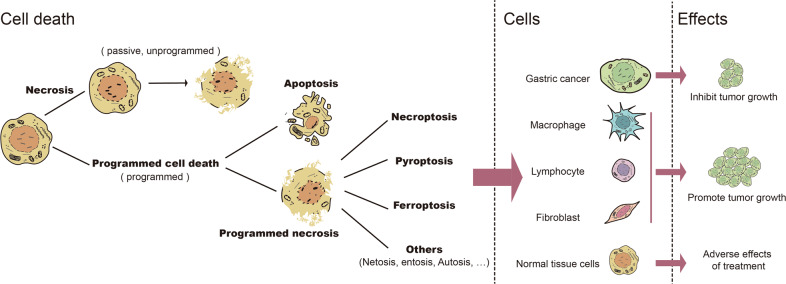
The relationship between cell death and gastric cancer. Left: The classification of cell death. Middle: Cells that undergo death in gastric cancer. Right: Effects triggered by different cell death in gastric cancer.

Cell death is a physiological process that maintains biological development and homeostasis of the internal environment. It is a common pathological mechanism that causes local or systemic inflammatory responses and triggers organ dysfunction and disease [13]. All life processes, including cell death, can be divided into passive and programmed processes based on whether they are regulated by proteins that are the executors of the life processes. The left side of Fig. 1 shows the classification of cell death divided into passive necrosis and PCD, and the latter is further divided into apoptosis and programmed necrosis, according to the different morphological characteristics of death. The two main morphological manifestations of cell death are apoptosis and necrosis. The former occurs with cell crumbling and intact cell membranes, with characteristic apoptotic vesicle formation, resulting in cell phagocytosis [14]. However, apoptosis does not induce an inflammatory response [14]. Unlike apoptosis, necrosis is associated with cell swelling, rupture of cell membranes, and collapse of organelles, which usually induce a local or even systemic inflammatory response [14]. Over the last two decades, studies have shown that programmed necrosis is widespread in cells. It is a significant class of cell death and a large family that includes necroptosis, pyroptosis, ferroptosis, and other less common and mechanistically unclear forms of programmed cellular necrosis (Fig. 1). A recent study in *Science* reported cell death caused by the binding of copper to lipid-acylated components of the tricarboxylic acid cycle, inducing lipid-acylated protein aggregation and loss of iron-sulfur cluster proteins, called cuproptosis, which is dependent on the mitochondrial respiratory chain and also a new form of programmed death [15].

Cell death is involved in the progression of gastric cancer at several levels. Inhibition of apoptosis is a cause of extensive proliferation of gastric cancer cells. In contrast, abnormal cell death of immune cells such as macrophages, lymphocytes regulated by tumor-secreted cytokines, and surface receptors may be vital for gastric cancer cells to escape the immune response [16, 17]. The increased death of normal gastrointestinal parenchymal, alveolar, and renal epithelial cells during radiotherapy and chemotherapy may be the main reason for the adverse effects of treatment [18]. The middle and right sides of Fig. 1 demonstrate the effects of several types of aberrant cell death on gastric cancer progression and treatment.

### Necrosis

As previously mentioned, necrosis is a passive form of cell death that is not regulated by any molecular pathway-related protein. Passive cell death is only influenced by physical and chemical laws, such as altered osmotic pressure, high temperature, high pressure, hypoxia, freezing, and mechanical shear forces [19]. It is characterized by the rupture of cell membranes and release of cell contents, a process that causes an inflammatory response [19]. Some anti-cancer drugs inhibit angiogenesis in the central tumor area to promote cellular hypoxia and depletion of tissue nutrients, thereby inducing cellular necrosis [20]. A large amount of content released by necrotic cells forms damage-associated molecular patterns which promote the inflammatory response [20]. These cascades of amplified inflammatory responses effectively inhibit tumor growth in the initial stages [21]. Unfortunately, the chronic inflammatory environment may alter tumor metabolism and promote the growth of non-necrotic tumor cells [21] by promoting the production of reactive oxygen species (ROS), secretion of cell growth factors, and activation of anti-apoptotic signaling pathways such as NK-κB [21]. This may explain, to some extent, the inconsistent efficacy of anti-angiogenic drug treatments in some patients. The inhibition of the chronic inflammatory response generated during tumor cell necrosis is a possible way to improve the efficacy of anti-cancer drugs.

### Apoptosis

Australian pathologist John Kerr discovered crumpled cell death without cell membrane rupture in 1956, and in 1972, he named it apoptosis and elaborated on the difference between apoptosis and necrosis [7]. In 1982, Horvitz et al. sequentially identified two mutant strains in nematodes resistant to apoptosis by genetic experiments, which they named abnormal cell death 3 (ced-3) and ced-4 [22]. In 1993, Horvitz’s student Junying Yuan cloned the nematode ced-3 sequence and at the same time discovered that ced-3 was a cysteine protein hydrolase with high homology to interleukin 1β-converting enzyme (ICE) in mammals and that overexpression of either ced-3 or ICE in rats induced apoptosis [23]. Later, ced-3 was found to be caspase-3 in mammals, which is the executive protein of apoptosis, whereas ced-4 is caspase-9 in mammals, which is an important protein in endogenous apoptosis [24, 25]. A large number of studies over the last three decades have resolved the specific mechanisms of apoptosis [24].

**Fig. 2:**
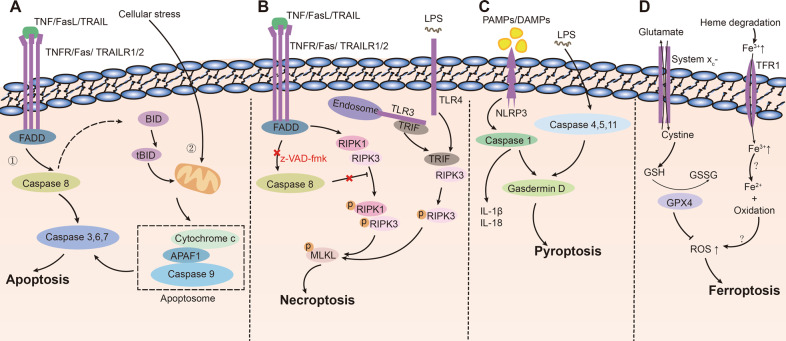
Molecular mechanisms of four common PCDs. **A** Molecular mechanisms of apoptosis. **B** Molecular mechanisms of necroptosis. **C** Molecular mechanisms of pyroptosis. **D** Molecular mechanisms of ferroptosis.

As shown in Fig. 2A, there are two main pathways of apoptosis: the extrinsic (①) and intrinsic pathways (②). In the exogenous activation pathway, some ligands, such as tumor necrosis factor (TNF), Fas, TNF-related apoptosis-inducing ligand (TRAIL), etc., activate death receptors (e.g., TNFR, Fas, TRAILR1/2) on the cell membrane, after which the intracellular structural domains of these death receptors recruit junctional molecules (e.g., fas-associated protein with death domain [FADD]). FADD further recruits pro-caspase-8 and activates it. Caspase-8 is a protein that can be self-activated by shearing and is considered the initiator protein of the exogenous apoptosis pathway [22]. Activated caspase-8 activates caspase-3, 6, 7 by cleaving pro-caspase-3, 6, and 7, which act as apoptotic execution proteins leading directly to apoptosis. Activation of caspase-3 further activates downstream proteins, such as poly (ADP-ribose) polymerase (PARP), a DNA repair enzyme that becomes active after cleavage by caspase-3 and has a direct effect on DNA [22]. In the endogenous activation pathway, the mitochondrial membrane becomes more permeable after damage from various intra- and extracellular stimuli, allowing the release of cytochrome c into the cytoplasm between the inner and outer mitochondrial membranes. Cytochrome c binds to apoptotic peptidase-activating factor 1 (APAF1) to recruit and cleave pro-caspase-9. Cytochrome c, APAF1, and activated caspase-9 form the apoptosome, which further activates caspase-3,6,7 [22]. However, exogenous and endogenous activation pathways of apoptosis do not exist independently. Activated caspase-8 cleaves BH3-Interacting Domain (BID) and forms tBID, tBID causes mitochondrial damage and cytochrome c release, which triggers the endogenous apoptotic pathway [22]. Overall, caspase-8 and caspase-9 are the initiating proteins of apoptosis, while caspase-3, 6, and 7 are important executive proteins involved in apoptosis. Activation of the exogenous apoptotic pathway is a series of responses triggered by the activation of death receptors, whereas the endogenous apoptotic pathway is triggered by mitochondrial damage. In addition to the previously mentioned TNFR, Fas, TRAILR1/2, IFNR, TLR, and DAI/ZBP1 are common death receptors. They are either localized at the cell membrane or endosome, and their activation induces the onset of apoptosis [26]. Notably, the death receptor DAI is localized in the cytoplasm and its ligand is not a protein or small-molecule substance, but DNA or RNA, which allows cells expressing this receptor to recognize exogenous nucleic acids when stimulated by microorganisms such as; viruses and bacteria, thus inducing apoptosis of the cells for pathogen removal [27]. Death domains are key to the activation of exogenous apoptotic pathways by death receptors such as DAI, and they can recruit FADD, TNF receptor-associated death domain and other junctional proteins [28]. One terminal of the junction protein is attached to a death domain-binding region, and the sequence at the other terminal binds to caspase and causes shearing and activation of caspases [28].

Apoptosis is regulated by checkpoints within the cell. As mentioned earlier, junctional proteins bind many proteins such as receptor-interacting serine/threonine-protein kinase 1 (RIPK1) to form Complex1 (also known as DISC, death-inducing signaling complex), but in normal cells, when DISC is formed, it triggers the activation of pro-survival signaling pathways such as the NF-κB pathway, which in turn inhibits apoptosis-related proteins [29]. Therefore, apoptosis can be induced in the laboratory only when both ligands (e.g., TNF) and inhibitors of pro-survival (e.g., CHX, ActD) are added to the cell culture media [29].

Apoptosis plays an irreplaceable role in embryonic development and throughout the lifespan of an organism. Excessive apoptosis may cause degenerative diseases, such as Alzheimer’s and other neurodegenerative diseases, while insufficient apoptosis is characteristic of many cancer cells [30]. Some cancer cells escape apoptosis caused by endogenous DNA damage and exogenous T cell killing. They also express ligands, such as programmed death-ligand 1 (PD-L1), to suppress the function of T cells and achieve immune escape [24]. In a review published by Rebecca et al. in 2011, five ways in which cancer cells evade apoptosis were described: (1) the reduced expression of death receptors in cancer cells, (2) a high expression of proteins that antagonize apoptosis (e.g., Bcl-2), and (3) Overexpression of proteins associated with inhibition of apoptosis (e.g., IAP); (4) A reduced expression of caspases and (5) p53 mutations [24]. Understanding these anti-apoptotic mechanisms in cancer cells makes it possible to find corresponding drugs that promote apoptosis and treat cancer. Chemotherapeutic drugs such as cisplatin promote apoptosis through bypass death receptors, Bcl-2 inhibitors, and IAP inhibitors. They effectively increase the level of apoptosis in cancer cells, and the use of drugs to activate caspases and bypass p53 contributes to apoptosis in cancer cells [24]. Research on apoptosis is important for the treatment of cancer.

### Necroptosis

In 1985, Scott Laster et al. found that rat fibroblast cell F-17 underwent apoptosis when stimulated by TNF, whereas mouse embryonic fibroblast cell L-M showed cellular necrosis after stimulation by TNF, suggesting that L-M cells may undergo a programmed death different from apoptosis [31]. However, this study did not receive much attention for more than a decade, mainly due to the widespread disbelief that necrosis could be regulated programmatically. It was not until 2000 that Tschopp et al. at the University of Lausanne, Switzerland, discovered that stimulation with Fas in some cells occurred in a PCD manner that exhibited a necrotic morphology independent of caspase-8 [32]. The authors found that when T cells express caspase-8, the cells undergo apoptosis when stimulated by death, whereas if T cells do not express caspase-8, they undergo programmed necrosis when stimulated by death, which is regulated by RIP (later named RIPK1) [32]. In 2005, Yuan et al. used a large-scale drug screen to find drugs that could inhibit RIPK1-mediated programmed necrosis, and they found that Necrostatin-1 (Nec-1) could inhibit TNF + z-VAD (inhibitor of caspases)-induced programmed necrosis in a variety of cells, and named this caspase-independent programmed necrosis necroptosis [33]. Later studies have confirmed that the target protein of Nec-1 is RIPK1 [34]. Over the last decade, studies have gradually elucidated the mechanism of necroptosis.

As shown in Fig. 2B, TNF, Fas, and other substances activate proteins with RHIM domains via death receptors. Only four proteins containing RHIM domains have been identified: RIPK1 (downstream of death receptor FADD), DAI (intracytoplasmic RNA/DNA ligand), and TRIF (downstream of TLR3 and TLR4, which identified LPS). These three proteins interact with the RHIM domain of RIPK3 (also having an RHIM domain) either separately or simultaneously to phosphorylate and activate mixed lineage kinase domain-like protein (MLKL). MLKL is an executive protein involved in necroptosis [29, 35]. After MLKL phosphorylation, its conformation changes and translocates to the cell membrane, leading to membrane disruption and necroptosis [35]. In summary, cells with death domains undergo both apoptosis and necroptosis. When activated by exogenous substances, whether apoptosis or necroptosis occurs depends on whether the cells’ caspases are inhibited and whether the cells express RIPK1 [35]. Similar to apoptosis, necroptosis is regulated by regulatory checkpoints such as pro-survival [29]. Therefore, the addition of TNF, z-VAD, and inhibitors of NF-κB to cells expressing both caspase-8 and RIPK1 induces necroptosis [29].

Unlike apoptosis, which is widespread in biological development, necroptosis is not involved in the developmental process of an organism [36]. Kaiser et al. found that apoptosis was inhibited in mouse embryos when caspase-8 was knocked out, which eventually died due to uncontrollable necroptosis, and the deletion of necroptosis rescued mouse embryo death caused by blocked apoptosis [37]. The embryos of mice with apoptosis- and necroptosis-related gene knockout can grow and develop; however, they develop lymphoid organ-related diseases as they age in adulthood, suggesting that necroptosis plays an irreplaceable role in the maturation of lymphocytes [37]. A study on lung cancer metastasis found that cancer cells activate endothelial cell necroptosis through APP and death receptor 6 (DR6) during metastasis and that necroptosis disrupts the integrity of the blood vessel wall, thus providing a pathway for cancer cells to metastasize [38].

### Pyroptosis

In 1989, the laboratories of Kostura MJ and Black RA each reported a new protein that could activate pro-IL-1β and named it ICE [39, 40]. ICE hydrolyzes pro-IL-1β between Asp at position 116 and Ala at position 117 to produce active IL-1β [39]. As mentioned earlier, Yuan et al. cloned ced-3, which is highly homologous to ICE, in the nematode in 1993, and they officially named the ICE/ced-3 family as caspases, ICE as caspase-1, in a paper published in *cell* in 1996 [23, 41]. A caspase-1-dependent mode of cell death accompanied by a large release of IL-1 was identified in the 1990s, but at the time, it was mistaken for apoptosis [42, 43]. It was not until 2000 that Brennan et al. found that macrophages infected with Salmonella experienced cell death, which was distinctly different from apoptosis; they showed necrotic cell morphology, and their occurrence was not dependent on caspase-3 activation but only on caspase-1 activation [44]. Subsequently, Boise and Collins, in a review of *Trends in Microbiology*, named this caspase-1-dependent programmed necrosis of cells with an accompanying inflammatory response pyroptosis [45]. Based on recent findings, in 2018, the Nomenclature Committee on Cell Death (NCCD) defined pyroptosis as regulated cell death dependent on the formation of plasma membrane pores by the gasdermin family of proteins, which is often, but not always, completed by the activation of inflammatory caspases.

The discovery of pyroptosis took a long time, and its molecular mechanism was fully elucidated in 2015 [46, 47]. As shown in Fig. 2C, when cells are stimulated by different bacteria or proteins, these pathogen-associated molecular patterns (PAMPs) are recognized by pattern recognition receptors (e.g., NLRP3) on the cell membrane, forming the inflammasome. Five types of inflammasome have been identified: NLRP1, NLRP3, NLRC4, IPAF, and AIM2 [48]. They all activate caspase-1, which has two roles: first, it cleaves gasdermin D to release the N-terminal, which leads to pyroptosis; second, it cleaves pro-IL-1β and pro-IL-18, allowing the maturation and release of IL-1β and IL-18. As shown in Fig. 2C, in a class of caspase-1-independent pyroptosis, intracellular LPS can be directly recognized by caspase-4,5,11, and activated caspase-4,5,11,which directly cleave Gasdermin D leading to pyroptosis. Also, this process induces inflammasome activation, followed by further caspase-1-mediated pyroptosis [48]. Thus, the key mechanism of pyroptosis is activating the gasdermin family of proteins [48]. In addition to gasdermin D, there are many other proteins in this family, such as gasdermin A, B, C, E, and DFNB59. With the exemption of DFNB58, several gasdermin proteins are predicted to have the ability to induce pyroptosis based on their structural domains [49].

Pyroptosis is distinct from apoptosis and necroptosis, and the study found that the deletion of the Gasdermin family of proteins did not affect development in mice [50]. In cancer immunotherapy, cytokine release syndrome (CRS) caused by CAR-T therapy is a serious complication that usually intensifies with increasing CAR-T efficacy. In a 2020 study, researchers found that CAT-T cells activate caspase-3/gasdermin E by activating granzyme B in tumor cells, thereby inducing tumor cell pyroptosis [51]. The killed tumor cells release factors such as ATP/HMGB, which are taken up by macrophages and induce intracellular macrophage caspase-1/gasdermin D activation, resulting in CRS [51]. Therefore, the clinical use of a specific inhibitor of gasdermin D for the activation of gasdermin E in tumor cells, and the blocking of gasdermin D, can prevent the occurrence of CRS while exerting CAR-T efficacy, thus improving the safety of treatment [51]. In addition, granzyme B from killer T cells directly cleaves gasdermin E and causes pyroptosis in tumor cells [52]. These pyroptosis-related studies provide ideas for cancer immunotherapy.

### Ferroptosis

The understanding of ferroptosis has come a long way. As early as the 1950s, researchers discovered that if some cells were deprived of amino acids, particularly cysteine, the cells showed some specific form of death, but this death was not defined or taken seriously at the time [53]. In the 1960s, it was discovered that excess accumulation of liposomal ROS in cells was involved in cell death due to nutrient deficiency [53]. ROS is a general term for reactive substances containing oxygen present in the body or natural environment [54]. In 2003, Stockwell et al. found that the small-molecule drug Erastin causes the death of cancer cells, which is different from the apoptosis, pyroptosis, and necroptosis that had been identified, and they suspected that this was a new form of PCD [55]. They found that RSL3 and RSL5 also cause cell death and that this is an iron-dependent process [56]. Four years later, they confirmed that drug-induced cell death could be inhibited by antioxidants [57]. In 2012, Brent et al. officially named this iron-dependent form of cell death, characterized by the accumulation of intracellular ROS ferroptosis [58].

Unlike other programmed deaths, ferroptosis, although programmatically regulated, occurs like a chain reaction caused by the excessive accumulation of metabolic substances without a clear initiating protein. As shown in Fig. 2D, under normal conditions, the cystine transporter protein system X_c_^−^ on the cell membrane transports cystine into the cell while transferring glutamate outside the cell. Cystine and glutamate are formed by enzymes to produce glutathione (GSH), which is assisted by glutathione peroxidase 4 (GPX4) to reduce intracellular oxygen radicals and prevent the formation of ROS. When any step of this process is blocked, such as the deficiency of cysteine, the inhibition of system X_c_^−^ or the inhibition of GPX4 causes an impairment of the processes, leading to the accumulation of ROS, which in the presence of free iron irreversibly causes damage to intracellular lipids and ultimately leads to the development of ferroptosis. However, when free iron is absent, the cell does not experience ferroptosis, even if there is a large accumulation of ROS [58]. The exact signaling pathway through which free iron induces cell death in ROS-accumulated cells remains unclear [58].

Ferroptosis remains a topic of interest for scientists, as the details of its regulatory mechanisms remain unknown. In 2019 *Nature*, Weiping Zou et al. found that γ-IFN released from CD8^+^ T cells promotes ferroptosis in cancer cells by inhibiting System X_c_^−^ and that the combination of PD-L1 and activators of ferroptosis enhances the killing effect of CD8^+^ T on tumor cells, thereby promoting tumor therapy [59]. In a 2020 study, the authors found significant mitochondrial damage, activation of autophagic lysosomes, and production of ROS in cancer cells after radiotherapy, suggesting that these cells experience ferroptosis [60]. These studies suggest that combining ferroptosis activators with multiple oncology therapies, such as immunotherapy and radiotherapy, may provide additional opportunities for future oncology treatments.

### Other programmed cell necrosis

In addition to the typical PCDs, such as apoptosis, pyroptosis, necroptosis, and ferroptosis, there are also less common PCDs such as lysosome-dependent cell death (LDCD), NET release-induced necrotic cell death (NETosis), and Entotic cell death (Entosis) [61]. It is possible that these uncommon PCDs are not independent programmed death processes but rather a concomitant phenomenon of the first four major cell deaths.

**Fig. 3:**
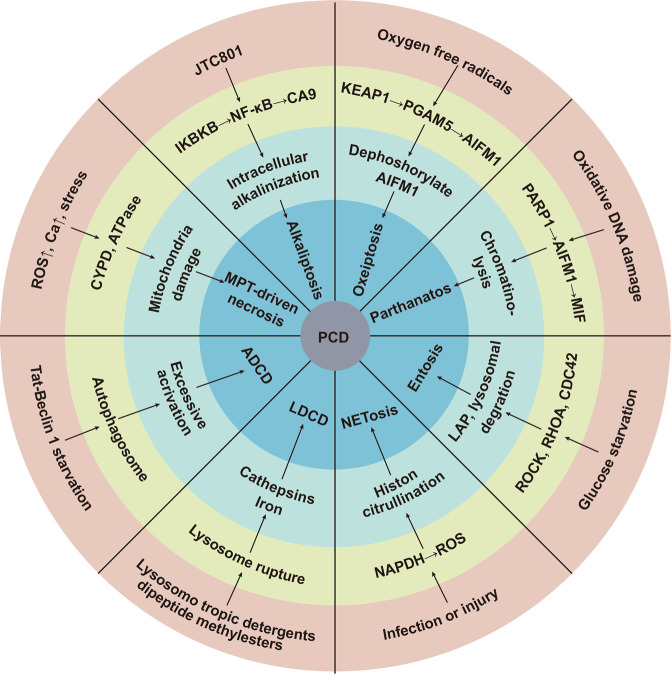
Molecular mechanisms of eight uncommon PCDs. MPT-driven necrosis, ADCD, LDCD, NETosis, Entosis, parthanatos, oxeiptosis and alkaliptosis.

The eight uncommon types of PCD are summarized in Fig. 3. MPT-driven necrosis refers to cell death induced by changes in the intracellular microenvironment, such as calcium overload in the mitochondria and ROS accumulation [62]. Autophagy-dependent cell death (ADCD) is caused by excessive activation of cellular autophagy: Tat-Beclin 1 is an autophagy-inducing peptide that fuses the protein amino acids of BECN1 and HIV Tat to induce ADCD [61]. Many factors that upregulate Tat-Beclin 1 have the potential to cause excessive activation of autophagy, which induces ADCD [61]. However, the current understanding of ADCD remains controversial: (1) Autophagy occurs only along with cell death, and there is no direct correlation between them; (2) Autophagy mediates the occurrence of some types of cell death, such as apoptosis, and (3) Autophagy-dependent cell death is a mechanism independent of apoptosis and necrosis [63]. Another type of PCD that is as controversial as ADCD is LDCD, which is mediated by hydrolases or calcium released from lysosomes and is characterized by the rupture of lysosomes [61]. As the exact relationship between autophagy and cell death and between lysosomes and cell death remains unclear, it is not possible to determine whether ADCD and LDCD are necroptosis, apoptosis, or ferroptosis caused by autophagy and lysosomal rupture or a completely new form of PCD [61]. NETosis is a NET-driven programmed death regulated by NADPH-mediated ROS production and histone citrullination [64]. NET is a net-like DNA-protein structure released by cells after infection [64]. Similar to NETosis, Entosis activates upstream ROCK and RHOA in cells to phagocytose and kill similar cells through LC3-related phagocytosis(LAP) and lysosomal degradation mediated by histone B [65]. PARP1-dependently regulated cell death (parthanatos) is oxidative stress-induced DNA damage: activated PARP1 binds to AIFM1 and mediates the translocation of AIFM1 from the mitochondria to the nucleus, causing partial chromosome lysis and cell death [66]. Parthanatos and apoptosis both involve PARP1 activation, but they differ as follows: (1) Parthanatos does not induce caspase activation; (2) Parthanatos occurs without the formation of apoptotic bodies; (3) Parthanatos is a programmed necrosis that is not accompanied by cell swelling, but only by cell membrane rupture [61]. Oxeiptosis is a novel programmed death induced by oxygen radicals that is not caspase-dependent but KEAP1-PGAM5-AIFM1-dependent [67]. Alkaliptosis is a form of cell death caused by IKBKB-mediated NF-κB signaling-dependent downregulation of carbonic anhydrase 9(CA9), a novel type of PCD driven by intracellular alkalinization [68]. The specific mechanisms of these PCDs are unclear, and their relevance to tumors has been little studied.

## Regulation of cell death in gastric cancer

Cell death is a complex biological process widely involved in gastric cancer progression. It is regulated by multiple factors both in vivo and in vitro and profoundly affects gastric cancer cells, immune cells, and the tumor microenvironment. In this section, we summarized the common cell death regulatory pathways in gastric cancer, which have positive implications for improving the malignant phenotype of gastric cancer.

### PI3K/AKT/mTOR

**Fig. 4:**
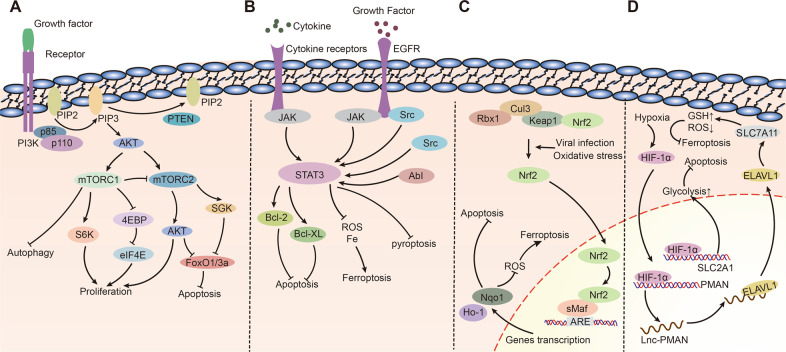
Common pathways regulating cell death in gastric cancer. **A** PI3K/AKT/mTOR. **B** JAK/STAT3. **C** Keap1/Nrf2. **D** Hypoxia/HIF-1α.

Phosphatidylinositol-3 kinases (PI3Ks) are intracellular phosphatidylinositol kinases with serine/threonine kinase activity and are divided into three classes depending on their structure and function [69]. The most widely studied of these is a class I PI3K, a heterodimer composed of a catalytic subunit (p110) and a regulatory subunit (p85) [69]. As shown in Fig. 4A, class I PI3Ks convert phosphatidylinositol-4, 5-bisphosphate (PIP2) to the second messenger phosphatidylinositol-3, 4, 5-trisphosphate (PIP3). There are two subclasses of class I PI3K: PI3K IA, which is activated by receptor protein tyrosine kinase (RTPK), and PI3K IB, which is activated by G protein-coupled receptors [69]. AKT is activated by the phosphorylation of PI3K. AKT is a threonine/serine protein kinase with two phosphorylation sites (Thr308 and Ser473) [70]. Phosphorylated AKT activates the mammalian target of rapamycin (mTOR) by directly phosphorylating mTOR or inactivating TSC2 [70]. mTOR is an evolutionarily highly conserved protein divided into mTOR complex1(mTORC1) and mTOR complex 2(mTORC2) [71]. mTORC1 regulates the expression of downstream eIF4E by activating S6K and inhibiting 4EBP, thereby inhibiting autophagy and promoting cell growth. In contrast, mTORC2 inhibits FoxO1/3a expression by activating SGK, thereby suppressing apoptosis [71]. In addition, AKT activation directly inhibits FoxO1/3a expression, thereby suppressing apoptosis [71]. Therefore, mTOR is an important regulator of apoptosis. In addition to PI3K/AKT, any signaling pathway that regulates mTOR, such as MAPK, Tp53, etc., will influence apoptosis. The tumor suppressor gene PTEN dephosphorylates PIP3 to form PIP2, thus negatively regulating this process [70].

The PI3K/AKT/mTOR signaling pathway regulates apoptosis in gastric cancer. Various drugs or compounds have been found to promote apoptosis of gastric cancer cells by inhibiting mTOR in gastric cancer. Salidroside and apolipoprotein C-II induce apoptosis in gastric cancer cells by inhibiting the PI3K/AKT/mTOR signaling pathway, thus inhibiting the progression of gastric cancer [72, 73]. Another study on ferroptosis-related long non-coding RNA (lncRNA) found that patients in the high-risk group had a better response to drugs targeting PI3K/AKT, WNT signaling, and the cytoskeleton, suggesting that aberrant activation of signaling pathways such as PI3K/AKT may inhibit ferroptosis in gastric cancer cells [74].

### JAK/STAT3

Signal transducer and activator of Transcription3 (STAT3) was one of the first oncogenes identified, and is involved in various biological processes such as cell growth, differentiation, and cell death, and is closely related to tumors [75]. There are three isoforms of STAT3: STAT3α, STAT3β, and STAT3γ, of which the most frequently studied is STAT3α, abbreviated as STAT3 [76]. STAT3 is the intersection of many signaling pathways activated by oncogenes, cytokines, and growth factors, of which the classical pathway is the JAK-STAT3 pathway. As shown in Fig. 4B, multiple cytokines or growth factors stimulate cytokine and growth factor receptors on the cell membrane, and these receptors recruit Janus kinases (JAK) in the cytoplasm and phosphorylate JAK [76]. Activated JAK changes its conformation and binds to the SH2 structural domain of STAT3, thereby phosphorylating STAT3 [76]. In addition to JAK, STAT3 is phosphorylated by non-receptor complex kinases in the cytoplasm, such as Src and AbI [77]. Phosphorylation of STAT3 causes a series of complex cascade reactions that affect multiple biological processes such as tumor cell proliferation, differentiation, cell death, and immune responses [78].

In gastric cancer, IL-26-activated STAT3 inhibits apoptosis by inducing the upregulation of the anti-apoptotic genes Bcl-2 and Bcl-XL and the oncogene c-Myc [79]. Many drugs that inhibit STAT3 activation promote apoptosis in gastric cancer cells. For example, CYT997 inhibits the JAK/STAT3 signaling pathway by inducing mitochondrial ROS accumulation, thereby promoting autophagy and apoptosis in gastric cancer cells [80]. Vorinostat also promotes apoptosis in gastric cancer cells by inhibiting the STAT3-IGF1R-HDAC3 signaling pathway [81]. In addition to the inhibition of apoptosis, the activation of STAT3 is associated with the inhibition of ferroptosis and pyroptosis. It was found that propofol increased the levels of ROS and free iron in gastric cancer cells, which led to ferroptosis, and overexpression of STAT3 inhibited propofol-induced ferroptosis [82]. Another study found that apatinib and cinobufagin encapsulated in a pH-responsive liposome induced pyroptosis and apoptosis in gastric cancer cells via the VEGFR2/STAT3 signaling pathway [82]. This new drug encapsulated by pH-responsive liposomes has better solubility and targeting ability compared with unencapsulated apatinib and cinobufagin, which improves drug effects and reduces adverse drug reactions [82]. These studies suggest that efficient STAT3 inhibitory drugs can help to induce gastric cancer cell death and treat gastric cancer.

### Keap1/Nrf2

Nrf2 is a member of the transcription factor family encoded by approximately 250 genes consisting of seven Neh structural domains that play an important role in the antioxidant response of the body [83]. Keap1 is a cullin3 (Cul3)-dependent bridging protein that assembles with Cul3 and Rbx1 to form a functional E3 ubiquitin ligase complex (Keap1-Cul3-Rbx1), which in turn regulates Nrf2 [84]. As shown in Fig. 4C, in normal cells, Nrf2 in the cytoplasm binds to the Keap1-Cul3-Rbx1 conformer, and non-functional Nrf2 is ubiquitinated and degraded by Keap1. However, when cells are stimulated by a viral infection or oxidative stress signals, Nrf2 dissociates from Keap1. Subsequently, it enters the nucleus, where it binds to small Maf (sMaf) proteins to form complexes that further bind to the promoter regions (ARE) of some genes and promote their transcription. Increased transcription of Ho-1 and Nqo1 resulted in increased translation of Ho-1 and Nqo1 in the cytoplasm, and their high expression in the cytoplasm inhibited the accumulation of ROS in the cell, thus suppressing ferroptosis [85].

A study at the First Hospital of China Medical University showed that ATF3 promotes ferroptosis by inhibiting the Keap1-Nrf2 axis, thereby increasing the sensitivity of gastric cancer cells to cisplatin [86]. Another study showed that silencing SIRT6 induced ferroptosis in gastric cancer cells by inducing inactivation of Keap1-Nrf2 and low expression of GPX4, thereby improving the sensitivity of gastric cancer cells to sorafenib [87]. It has also been shown that ROS accumulation activates the Keap1-Nrf2 signaling pathway in gastric cancer cells, thereby inhibiting apoptosis in gastric cancer cells [88]. However, LCT-3d induces oxidative stress and apoptosis in gastric cancer cells by upregulating death receptor 5, a process unaffected by Nrf2 activation [88]. This suggests that the inhibitory effect of the Nrf2 pathway on apoptosis is weak and not readily apparent when other proapoptotic signals are activated. However, when Nrf2 signaling is heavily degraded by ubiquitination in the presence of BDH2, gastric cancer cells undergo apoptosis due to the accumulation of ROS [89]. These findings suggest that the Keap1-Nrf2 signaling pathway plays an important regulatory role in apoptosis and ferroptosis induced by the oxidative stress response.

### Hypoxia/HIF-1α

Hypoxia is a common feature of solid tumors, and a higher degree of hypoxia is closely associated with a poor prognosis and reduced survival rate. Hypoxia induces the upregulation of hypoxia-inducible factor (HIF-1) in tumor cells [90]. HIF is a heterodimer of HIF-1α and HIF-1β. After entering the nucleus, HIF-1α regulates the expression of more than 300 proteins and has a profound effect on tumor metabolism, proliferation, migration, and cell death [90]. As shown in Fig. 4D, hypoxia-induced high expression of HIF-1α in the cell was activated and entered the nucleus to bind to the PMAN gene, thereby upregulating the level of lnc-PMAN. Lnc-PMAN Translocates ELAVL1 from the nucleus to the cytoplasm, and cytoplasmic ELAVL1 increases the stability of SLC7A11 mRNA. High cytoplasmic expression of SLC7A11 increases GSH expression and decreases ROS levels, thereby inhibiting ferroptosis [91]. Another study showed that DpdtbA induced ferroptosis in gastric cancer cells by activating the P53 and PHD2/HIF-1α signaling pathways in gastric cancer [92]. Hypoxia-induced HIF-1α activation also promotes glycolysis by promoting SLC2A1, which inhibits apoptosis [93, 94]. These results suggest that high expression of HIF-1α inhibits the apoptosis and ferroptosis of gastric cancer cells, and an effective HIF-1α inhibitor can promote gastric cancer cell death.

### Non-coding RNA

In recent years, research on non-coding RNAs and gastric cancer has been in full swing, and a large number of circRNAs, lncRNAs, and miRNAs have been identified, which form a complex regulatory network by cross-talk with each other to regulate various biological behaviors in gastric cancer. All the signaling pathways summarized earlier may be regulated by specific non-coding RNA networks. Apoptosis is the most studied model of cell death in gastric cancer, and there are hundreds of non-coding RNA pathways that regulate apoptosis in gastric cancer cells through competitive endogenous RNA mechanisms, and we do not give detailed examples here. In this section, we summarized only the non-coding RNAs that regulate programmed necrosis in gastric cancer.

Table 1 shows several non-coding RNAs that regulate programmed cell death in gastric cancer cells [91, 95–98]. The low expression of lncRNA SNHG1 in gastric cancer leads to the upregulation of miR-21-5p expression to suppress the level of downstream TLR4, thereby inhibiting the activation of intracellular necroptosis [95]. High expression of ADAMTS9-AS2 enhanced the sensitivity to cisplatin, which was mainly due to ADAMTS9-AS2 sponging miR-223-3p to promote the expression of NLRP3, thus promoting pyroptosis in gastric cancer cells [96]. Hypoxia-induced lncRNA-PMAN increases the stability of SLC7A11 by facilitating the translocation of ELAVL1 from the nucleus to the cytoplasm, thereby inhibiting ferroptosis in gastric cancer cells [91]. The lncRNA BDNF-AS is highly expressed in gastric cancer and affects the ubiquitination of VDAC3 via WDR5/FBXW7, thereby inhibiting ROS accumulation and ferroptosis in cells [97]. Circ_0000190/miR-382-5p/ZNRF3 regulates ferroptosis in gastric cancer [98].

**Table 1:** Non-coding RNAs that regulate programmed necrosis in gastric cancer cell.

Programmed necrosis	RNA	Dyregulate	miRNA	Pathway	Ref.
necroptosis	lncRNA SNHG1	downregulate	miR-21-5p	TLR4	[93]
pyroptotic	ADAMTS9-AS2	downregulate	miR-223-3p	NLRP3	[94]
ferroptosis	lncRNA-PMAN	upregulate		ELAVL1	[89]
	lncRNA BDNF-AS	upregulate		WDR5/FBXW7	[95]
	circ_0000190	downregulate	miR-382-5p	ZNRF3	[96]

Table 2 shows several programmed necrosis-associated non-coding RNAs as predictive models for gastric cancer [99–107]. Most of these models are obtained by bioinformatics prediction of upstream regulatory lncRNAs of the corresponding cell death mode, combined with the expression levels and survival information of these molecules in the TCGA database in gastric cancer; ROC curves are performed to determine the prognosis of this combined mode, and then validated in clinical samples to screen the most effective prognostic prediction model. Among them, the number of necroptosis-, pyroptosis-, and ferroptosis-related lncRNA prognostic models in gastric cancer were two, one, and six, respectively (Table 2).

**Table 2:** Programmed necrosis-associated non-coding RNA as a predictive model for gastric cancer.

Programmed necrosis	RNA	AUC of ROC	Ref.
necroptosis	**16 lncRNAs**: LINC01829, LINC02657, RNF139-AS1, FRMD6-AS2, AGBL5-IT1, AC116914.1, AC005165.1, AL353804.2, AC004596.1, AL355574.1, AC012409.3, AC124067.4, AC015813.1, AP001189.3, AL133245.1, AC069549.1	0.770	[97]
	**12 lncRNAs**: REPIN1-AS1, UBL7-AS1, LINC00460, LINC02773, CHROMR, LINC01094, FLNB-AS1, ITFG1-AS1, LASTR, PINK1-AS, LINC01638, PVT1	0.748	[98]
pyroptotic	**11 lncRNAs**: AL353804.1, AC147067.2, AP001318.2, RRN3P2, UBL7-AS1, AC018752.1, ACTA2-AS1, AL121772.1, AC005332.4, HAGLR, AC245041.2	0.850	[99]
ferroptosis	**20 lncRNAs**: AC114271.1, AC147067.2, AL353796.1, AC104958.1, AC087521.1, AL590705.3, AC068790.7, AC090772.1, LINC01094, AC007405.3, AC083902.1, LINC00460, AC005165.1, AC048382.2, AC106782.5, STX18-AS1, AL355574.1, CYMP-AS1, AC006547.1, LINC02696	0.947	[100]
	**17 lncRNAs**: ENSG00000249835.2, ENSG0000023671 9.2, ENSG00000250241.4, ENSG00000240661.1, ENSG0000026 2061.4, ENSG00000229656.5, ENSG00000175746.6, ENSG00000 248599.1, ENSG00000254333.1, ENSG00000247134.5, and ENSG00000248362.1, ENSG00000234449.2, ENSG0000023 9513.4, ENSG00000265334.1, ENSG00000267201.1, ENSG 00000273293.1, ENSG00000230107.1	0.751	[101]
	**4 lncRNAs:** AP003392.1, AC245041.2, AP001271.1, BOLA3-AS1	0.636	[102]
	**12 lncRNAs**: AC026368.1, CFAP61-AS1, AC090772.1, LINC00449, AC005165.1, LINC01614, AL356215.1, REPIN1-AS1, LASTR, LINC00460, AC015712.1, PVT1.	0.734	[103]
	**6 lncRNAs**: STX18-AS1, MIR99AHG, LINC01197, LINC00968, LINC00865, LEF1-AS1	0.640	[104]
	**3 lncRNAs**: P000695.2, AL365181.3, LINC01615	0.670	[105]

The bold values represent the number of lncRNAs in each gastric cancer prediction model.

### ADCD and gastric cancer

As mentioned earlier, since the scientific community is still inconclusive about whether ADCD is an independent PCD, we only briefly summarized the effect of ADCD on gastric cancer in this section. Many studies on Chinese medicine and tumors have identified herbs that induce ADCD in gastric cancer cells. Naringin flavonoid promotes autophagy in AGS cells by inhibiting the PI3K/Akt/mTOR signaling pathway [108]. Activated autophagic vesicles fuse with swollen mitochondria and ribosomes, further activating autophagic lysosomes, which release large amounts of histone D into the cytoplasm, causing cell digestion and death [108]. Kaempferol promotes ADCD by activating IRE1-JNK-CHOP to promote LC3-I to LC3-II conversion and p62 downregulation [109]. Another study found that cinnamaldehyde promotes gastric cancer cell death by activating the PERK-CHOP signaling pathway and affecting calcium ion homeostasis in gastric cancer cells. This effect can be blocked by autophagy inhibitors, suggesting that cinnamaldehyde inhibits gastric cancer by inducing ADCD in gastric cancer cells [110]. These herb-related studies have opened our eyes to the possibility of anti-cancer treatment by identifying more effective drugs to induce ADCD in gastric cancer cells. However, not all factors that activate autophagy can induce ADCD. In some cases, activation of autophagy promotes the clearance of harmful intracellular substances by tumor cells, and such autophagy protects tumor cells from cell death. A study on *Autophagy* found that the lncRNA EIF3J-DT upregulates ATG14 expression by sponging miR-188-3p, thereby activating autophagy to induce chemotherapy resistance in gastric cancer cells [111]. These results suggest that proper autophagy may protect tumor cells, whereas over-activated autophagy may induce ADCD in tumor cells. Therefore, there is still a long way to go to find drugs that efficiently induce ADCD in gastric cancer cells.

## Cell death and the tumor microenvironment in gastric cancer

In the previous section, we reviewed the direct effects of cell death on gastric cancer cells in detail, and we will continue to explore the effects of cell death occurring in the tumor microenvironment.

Exosomes released from tumor-associated macrophages were taken up by gastric cancer cells, promoting MAPK phosphorylation and high expression of PD-L1 in gastric cancer cells [112]. The activation of the MAPK signaling pathway directly inhibited apoptosis of gastric cancer cells. In contrast, the binding of PD-L1 to PD-1 on the surface of T cells initiated the PCD of T cells, which finally allowed gastric cancer cells to obtain immune escape [112]. Another study showed that gastric cancer cells with high FAS-L expression were more likely to induce apoptosis of tumor-infiltrating lymphocytes (TIL), thus achieving immune escape of gastric cancer cells [113, 114]. High TRAIL in gastric cancer cells also promotes apoptosis of TIL by prompting gastric cancer cells to bind to TIL [115]. The number and activity of NK cells in gastric cancer tissues are independent risk factors affecting the prognosis of patients with advanced gastric cancer [116]. A study found that prostaglandin E2 secreted by gastric cancer cells inhibited the proliferation of NK cells and promoted the apoptosis of NK cells [116]. These results suggest that gastric cancer induces PCD in immune cells through the high expression of various cell membrane surface ligands, release of exosomes, or secretion of cytokines and chemicals to achieve autoimmune escape. Identifying effective immune checkpoint antibodies or inhibitors is a reliable way to enhance the immune cell response to tumors.

A study at the McGill University Health Center in Canada found that many malignancies, including gastric cancer, develop a tumor microenvironment that induces NETosis in neutrophils. The large number of DNA fibers released by neutrophils after NETosis constitutes a network that wraps around the tumor cells, and this network protects them from immune recognition and clearance as they migrate, thereby facilitating the metastasis of malignant tumors [117]. This is an interesting form of immune escape for malignant tumor cells, but there are no studies on the mechanisms in the tumor microenvironment that contribute to neutrophil NETosis.

## Prospects and conclusions

Our study explored the relationship between thirteen types of cell death and gastric cancer to improve the clinical management of gastric cancer. In this section, we reviewed the available therapeutic options for gastric cancer and looked at possible directions for targeting the cell death pathway.

As shown in Fig. 1, the effect of cell death on gastric cancer is mainly reflected in three aspects: (1) Cell death in gastric cancer induced by various factors inhibits the proliferation of gastric cancer; (2) abnormal cell death of macrophages, lymphocytes, and fibroblasts causes immune escape and drug resistance of gastric cancer, thus promoting the progression of gastric cancer; (3) The death of normal cells induced by various regimens, such as radiotherapy, chemotherapy, and immunotherapy, is the main reason for treatment-related adverse reactions. Therefore, the search for effective anti-gastric cancer treatment from the perspective of cell death will also focus on these three aspects.

Many anti-gastric cancer drug studies have focused on inducing gastric cancer cell death. Salidroside and apolipoprotein C-II induce apoptosis in gastric cancer cells by inhibiting the PI3K/AKT/mTOR signaling pathway, thus inhibiting the progression of gastric cancer [72, 73]. CYT997 inhibits the JAK/STAT3 signaling pathway by inducing mitochondrial ROS accumulation, thereby promoting autophagy and apoptosis in gastric cancer cells [80]. Another study showed that DpdtbA induced ferroptosis in gastric cancer cells by activating the P53 and PHD2/HIF-1α signaling pathways in gastric cancer [92]. However, drug resistance and serious adverse reactions are two major problems that urgently need to be solved in clinical chemotherapy. As mentioned earlier, cisplatin and paclitaxel inhibit ferroptosis by promoting the secretion of miR-522 from tumor-associated fibroblasts, causing chemoresistance in gastric cancer cells [118]. In the future, the search for new drugs and the combined use of multiple drugs is expected to solve the situation that changes in the tumor internal environment and inhibition of cell death caused by a single drug for a long time. This will reduce the occurrence of chemotherapy resistance events. Programmed death 1 (PD-1) proteins are a class of co-inhibitory receptors on the surface of T cells [119]. Many malignancies expressing the PD-1 ligand PD-L1 can induce cell death of T cells through PD-1/PD-L1 binding, causing the immune escape of tumor cells [119]. Many immunotherapies are dedicated to finding immune checkpoint inhibitors, such as pembrolizumab and trastuzumab, to block tumor cell-induced cell death of lymphocytes, thus addressing the problem of tumor immune escape [120]. Finding new immune checkpoint inhibitors or antibodies or inhibitors of cell death signaling pathways in lymphocytes, fibroblasts, and macrophages is an effective way to reduce tumor immune escape and stimulate the anti-cancer response of the autoimmune system in the future. In addition to the therapeutic effect, the drug’s safety is also important in determining its suitability for long-term clinical application and patient adherence. Many adverse reactions of drugs for gastric cancer, such as bone marrow suppression and gastrointestinal reactions, are caused by the death of bone marrow, gastric mucosal epithelial, and intestinal epithelial cells [121, 122]. Identifying new drugs that induce tumor cell death with high specificity and combining the application of existing anti-cancer drugs with drugs that specifically inhibit cell death in the gastrointestinal tract and bone marrow are effective ways to mitigate the adverse effects of anti-cancer treatment in the future.

In conclusion, research on gastric cancer and cell death is increasing rapidly. This paper provides a detailed review of the multiple cell death modalities that influence gastric cancer progression and have profound effects on gastric cancer proliferation, invasion, migration, and immune response by acting directly on gastric cancer cells or the tumor microenvironment. Their study helps us to understand the mechanisms of gastric cancer progression more deeply so that we can find effective drugs or treatments to promote gastric cancer cell death, inhibit cell death of immune cells, and ultimately improve gastric cancer treatment.

## Data Availability

This is a review, and all data are included in the manuscript.
